# Publishing the data behind the data (Show me yours, I'll show you mine)

**DOI:** 10.14814/phy2.12186

**Published:** 2014-10-29

**Authors:** Gareth Leng

**Affiliations:** 1 Centre for Integrative Physiology, School of Biomedical Sciences, University of Edinburgh, Edinburgh, UK

## Introduction

Since the inception of *Physiological Reports*, the Editorial Board, together with our owners, The Physiological Society and the American Physiological Society, have been debating how to develop this journal in ways to better serve our community and our science.

The integrity of scientific journals was once safeguarded by academic communities – either directly, by the ownership and management of journals by learned societies, or indirectly, by academic institutional decisions about library subscriptions. The explosive growth of open access journals, with the entry of new for‐profit publishers, has threatened to undermine this model; it is now clear that some new open access journals are little more than vehicles for vanity publication with ineffective peer review (Bohannon 2013). This is perhaps the predictable outcome of a publication model where every paper accepted means a profit for the publisher, and every paper rejected is a cost. It is also why *Physiological Reports* has a robust peer review policy and encompasses the publishing ethos and standards of our two physiological societies.

The concerns about integrity not only touch the new open access journals; they also extend to the most established of journals (Nature 2013a,b). Most prominently, it was recently reported that the biotechnology firm Amgen tried to confirm findings published in 53 ‘landmark’ preclinical cancer studies; scientific findings were confirmed in only six cases (Begley and Ellis 2012).

One natural step to protect the integrity of the scientific literature is to require that, at the time of publication, authors make all of the data underlying the findings described in their manuscript fully available, without restriction (PLOS 2014). The open access revolution is fast becoming an open data revolution – one with massive implications for how we record our science.

This is a development that we wholeheartedly support, not only in the interests of safeguarding the integrity of published science but also because such data are potentially important for secondary use – for computational modeling, for meta‐analysis, and for secondary statistical analysis.

*Physiological Reports* intends to be at the forefront of this change. Over the coming year, we will develop ways to facilitate deposition by authors of the data behind their published summaries. Our approach will be pragmatic – we must *realize* the value inherent in data sets by making them openly available, but not imposing unrealistic requirements on authors that may add a major burden while serving no real secondary purpose. For data to be useful, they must be interpretable in the form in which they are deposited, and this requires some standardization of metadata, and requires that the metadata be searchable. Accordingly, we must evolve our policies with a view to best serving identified secondary uses – and it is this that must ultimately determine the forms of storage and metadata that will be most appropriate.

Accordingly, we will evolve our policies in discussion with *The Physiological Society* and *The American Physiological Society, and our publisher Wiley,* and in light of feedback from authors and editors.

As a start in this process, we can now offer authors the ability to submit as supplementary data the Excel spreadsheets that underlie their figures and tables. An example of how published papers can be enriched in this way is published in the October issue of *Physiological Reports* (Leng et al. 2014).

The direction of travel is clear and set: to enhance the quality of *Physiological Reports* for the greater benefit to science and the community we serve.
